# Vaccination response to tetanus toxoid and 23-valent pneumococcal vaccines following administration of a single dose of abatacept: a randomized, open-label, parallel group study in healthy subjects

**DOI:** 10.1186/ar2174

**Published:** 2007-04-10

**Authors:** Lee Tay, Francisco Leon, George Vratsanos, Ralph Raymond, Michael Corbo

**Affiliations:** 1 Clinical Discovery, Bristol-Myers Squibb, PO Box 4000, Princeton, NJ 08543-4000, USA; 2 Clinical Development, Inflammatory Diseases, MedImmune, 1 MedImmune Way, Gaithersburg, MD 20878, USA; 3 Global Clinical Research, Immunology, PO Box 4000, Bristol-Myers Squibb, Princeton, NJ 08543-4000, USA; 4 Global Biometric Sciences, PO Box 4000, Bristol-Myers Squibb, Princeton, NJ 08543-4000, USA

## Abstract

The effect of abatacept, a selective T-cell co-stimulation modulator, on vaccination has not been previously investigated. In this open-label, single-dose, randomized, parallel-group, controlled study, the effect of a single 750 mg infusion of abatacept on the antibody response to the intramuscular tetanus toxoid vaccine (primarily a memory response to a T-cell-dependent peptide antigen) and the intramuscular 23-valent pneumococcal vaccine (a less T-cell-dependent response to a polysaccharide antigen) was measured in 80 normal healthy volunteers. Subjects were uniformly randomized to receive one of four treatments: Group A (control group), subjects received vaccines on day 1 only; Group B, subjects received vaccines 2 weeks before abatacept; Group C, subjects received vaccines 2 weeks after abatacept; and Group D, subjects received vaccines 8 weeks after abatacept. Anti-tetanus and anti-pneumococcal (Danish serotypes 2, 6B, 8, 9V, 14, 19F and 23F) antibody titers were measured 14 and 28 days after vaccination. While there were no statistically significant differences between the dosing groups, geometric mean titers following tetanus or pneumococcal vaccination were generally lower in subjects who were vaccinated 2 weeks after receiving abatacept, compared with control subjects. A positive response (defined as a twofold increase in antibody titer from baseline) to tetanus vaccination at 28 days was seen, however, in ≥ 60% of subjects across all treatment groups versus 75% of control subjects. Similarly, over 70% of abatacept-treated subjects versus all control subjects (100%) responded to at least three pneumococcal serotypes, and approximately 25–30% of abatacept-treated subjects versus 45% of control subjects responded to at least six serotypes.

## Introduction

Treatment with abatacept has demonstrated efficacy in patients with active rheumatoid arthritis (RA) and an inadequate response to methotrexate, and in those with an inadequate response to anti-TNF therapy [1-3]. Abatacept is a soluble fusion protein consisting of the extracellular domain of human cytotoxic T-lymphocyte-associated antigen-4 linked to the Fc (hinge, CH2 and CH3 domains) portion of human IgG_1_, which has been modified to be noncomplement fixing. Abatacept is the first in a class of agents for the treatment of RA that selectively modulates the CD80/CD86:CD28 co-stimulatory signal required for full T-cell activation [4]. Activation of T cells usually requires two signals from antigen-presenting cells [5,6]. The first signal is mediated through the T-cell receptor via an interaction with major histocompatibility complex-presented peptide antigen [6]. The second, or co-stimulatory, signal is delivered following the engagement of CD80/CD86 on antigen-presenting cells with a cognate receptor, CD28, on the surface of the T cell [6,7]. Abatacept, a selective co-stimulation modulator, inhibits CD28-dependent T-cell activation by binding to CD80 and CD86 [4].

The impact of abatacept on humoral responses to two T-cell-dependent neoantigens, bacteriophage X174 and keyhole limpet hemocyanin, was previously evaluated in psoriasis patients treated with abatacept [8]. While the responses to these neoantigens were reduced, the primary response to these T-cell-dependent antigens was not completely blocked. In addition, tertiary and quaternary responses were restored following discontinuation of abatacept administration, demonstrating that tolerance to these neoantigens was not induced [8].

In the present article we describe the effect of a single dose of abatacept on the humoral response in healthy subjects to two vaccines, tetanus toxoid vaccine and 23-valent pneumococcal vaccine. This study was carried out in normal healthy subjects in order to evaluate the effects of abatacept on the response to therapeutic vaccines in intact immune systems before evaluating the response in RA patients. Patients with active RA may not have normal immune function parameters, and often receive background disease-modifying antirheumatic drugs, many of which are immunosuppressive. It was intended that data from this study would guide the design of other studies evaluating vaccine responses in patients with RA. These critical studies in 'real-world' RA patients are ongoing. In addition, the effect of abatacept upon two different types of antigen response was evaluated. The tetanus toxoid vaccine comprises a peptide antigen, and, since most individuals in the United States have been vaccinated with tetanus toxoid, the response measured in this study can be considered a T-cell-dependent memory response. Polysaccharides, however, are able to elicit responses in the absence of T-cell help, although the magnitude of the response is reduced under those circumstances [9-11]. The response to pneumococcal vaccine measured in the present study is therefore not entirely T-cell independent, or the response is less T-cell dependent. Finally, as a normal humoral response to T-cell-dependent antigens peaks at around 2 weeks [12], we also analyzed the impact on humoral response of the timing of vaccination relative to abatacept administration.

## Materials and methods

### Study design

**Figure 1:**
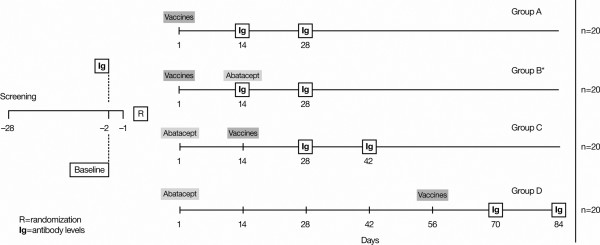
Patient disposition from enrollment to completion of the trial. *Abatacept administered after immunoglobulin (Ig) determination at day 14.

This open-label, parallel-group, controlled study was conducted at three study centers in the United States. Subjects were randomized to one of four treatment groups (Figure 1).

Group A (control group) subjects received separate 0.5 ml intramuscular (i.m.) injections of tetanus toxoid and 23-valent pneumococcal vaccines on day 1 without abatacept.

Group B subjects (vaccines 2 weeks before abatacept) received separate 0.5 ml i.m. injections of tetanus toxoid and 23-valent pneumococcal vaccines on day 1, followed 14 days later by a single intravenous (i.v.) dose of 750 mg abatacept. Serum samples were collected prior to the abatacept infusion on study day 14 and 14 days later on study day 28.

Group C subjects (vaccines 2 weeks after abatacept) received a single i.v. dose of 750 mg abatacept on day 1, followed 14 days later by separate 0.5 ml i.m. injections of tetanus toxoid and 23-valent pneumococcal vaccines. Serum samples were obtained on study day 14 prior to vaccinations and at 14 and 28 days after the vaccinations (study days 28 and 42, respectively).

Group D subjects (vaccines 8 weeks after abatacept) received a single i.v. dose of 750 mg abatacept on day 1, followed 56 days later by separate 0.5 ml i.m. injections of tetanus toxoid and 23-valent pneumococcal vaccines.

Serum samples were obtained for subjects of Groups A and B at study days 14 and 28, for Group C subjects at study days 28 and 42, and for Group D subjects at study days 70 and 84.

Healthy male or female subjects (aged 18–65 years inclusive) with a body weight ≥ 60 kg and ≤ 100 kg were enrolled. Subjects were excluded if they had received any live vaccine within the prior 4 weeks, had received a tetanus booster or pneumococcal vaccine within 5 years or if they had baseline anti-tetanus antibodies below clinically detectable levels. Anti-tetanus and anti-pneumococcal (Danish serotypes 2, 6B, 8, 9V, 14, 19F and 23F) antibody titers were measured by ELISA at 14 and 28 days after vaccination by a central laboratory. Abatacept serum concentrations were measured at the same time as the antibody titers were determined.

This study was carried out in accordance with the ethical principles of the Declaration of Helsinki and was approved by Institutional Review Boards. All subjects gave informed consent.

### Drug administration and vaccination

Abatacept 750 mg was administered over 30 minutes by i.v. infusion using a calibrated, constant-rate infusion. Tetanus toxoid vaccine (Aventis Pasteur Inc., Swiftwater, PA, USA) and 23-valent pneumococcal vaccines (Merck & Co Inc., Whitehouse Station, NJ, USA) were administered separately via i.m. injection in either the deltoid or the lateral mid-thigh.

### Abatacept and antibody assays

Serum samples were used to determine antibody levels. The assay to quantify IgG anti-tetanus toxoid antibody levels was based on a previously described methodology [13]. The assay to quantify IgG anti-pneumococcal antibody levels was based on the Procedures of the World Health Organization Pneumococcal Serology Reference Laboratories at the Institute of Child Health, University College, London, UK, and on the procedures of the Department of Pathology, University of Alabama at Birmingham, AL, USA [14]. All analyses were carried out at the Center for Vaccine Research and Development, St Louis University Health Sciences Center, St Louis, MO, USA.

The antibody response against tetanus toxoid vaccine was expressed as absolute titers of antibodies. Serum samples for the quantification of abatacept were collected at baseline, prior to vaccinations, and at the time when the samples were collected for antibody determinations [15].

### Safety assessments

Subjects were monitored for adverse events (AE), serious AE and vital signs prior to dosing with abatacept and upon discontinuation.

### Statistical methods

All subjects in all four groups were included in the safety analysis. Geometric means and the percentage of the coefficient of variation were reported for antibody concentrations. For each antibody, point estimates and 95% confidence intervals were constructed for the geometric mean changes from prevaccination to postvaccination antibody levels. These constructions were from the results of repeated-measures analyses of covariance on the natural logarithm of the antibody levels, with the treatment group and the study day as factors and the log of the baseline (prevaccination) antibody level as the covariate. For each antibody, point estimates and 95% confidence intervals for the prevaccination to postvaccination changes on the log scale were exponentiated to obtain estimates for geometric means and ratios of geometric means (fold increase) on the original scale. A twofold or higher increase above the baseline levels of specific antibodies was considered a clinically significant or positive immune response against tetanus toxoid and to each of the seven chosen serotypes of the 23-valent pneumococcal vaccine [16,17].

## Results

The baseline demographics and clinical characteristics of the 80 subjects enrolled in this study were similar across the four groups. The mean age of subjects was 34–36 years (Table 1). Of the 80 subjects, 77 (96%) completed treatment and three (4%) discontinued early from the study.

**Table 1:** Subject demographics

Characteristic	Group A (vaccines alone on day 1) (*n *= 20)	Group B (vaccines 2 weeks before abatacept) (*n *= 20)	Group C (vaccines 2 weeks after abatacept) (*n *= 20)	Group D (vaccines 8 weeks after abatacept) (*n *= 20)
Age (years)				
Mean	34	34	34	36
Standard deviation	12	13	11	13
Range	18–55	18–56	19–56	20–56
Gender (*n *(%))				
Male	10 (50)	8 (40)	10 (50)	11 (55)
Female	10 (50)	12 (60)	10 (50)	9 (45)
Race (*n *(%))				
White	15 (75)	12 (60)	14 (70)	11 (55)
Black	5 (25)	8 (40)	6 (30)	8 (40)
Other	0	0	0	1 (5)

Overall, 59 AE were experienced by 29 subjects (49.2%) treated with abatacept, compared with 25 AE reported in 13 subjects (65.0%) who did not receive abatacept (Group A, control group). The most frequently reported AE in Group A subjects were injection-site pain (50.0%), headache (10.0%) and pharyngolaryngeal pain (10.0%). The most frequently reported treatment-emergent AE in the abatacept-treated groups were headache (20.3%), injection-site pain (10.2%) and viral infection (10.2%).

One subject (1.7%) in Group D experienced a serious adverse event of generalized urticaria 5 minutes after the end of the first abatacept infusion, which re-occurred at 90 minutes postinfusion. The investigator reported the event as moderate in intensity and probably related to the study drug. The subject was treated with epinephrine and diphenhydramine, remained hospitalized overnight for observation and was discharged on study day 2. The event was completely resolved by study day 3.

### Abatacept serum concentration levels

The observed abatacept serum concentrations levels were consistent with the dose of abatacept administered and its relative timing.

Subjects randomized to Group A (control group, vaccines only) did not receive abatacept, as reflected in Table 2. Subjects randomized to Group B received vaccines 2 weeks prior to treatment with abatacept. In this group, serum samples were collected prior to the abatacept infusion on study day 14 and on study day 28. This is reflected in serum concentrations below the lower limit of quantification on day 14, and a mean serum concentration of 28.6 μg/ml on study day 28.

**Table 2:** Abatacept serum concentration levels determined 14 and 28 days after vaccination

Group	Baseline (μg/ml)	14 days after vaccination (μg/ml)	28 days after vaccination (μg/ml)
Group A^a ^(vaccines alone on day 1)	N/A	N/A	N/A
Group B^b ^(vaccines 2 weeks before abatacept)	N/A	N/A	28.6 (26)
Group C (vaccines 2 weeks after abatacept)	N/A	12.5 (19)	6.1 (20)
Group D (vaccines 8 weeks after abatacept)	N/A	1.3 (56)	0.4 (106)

Data presented as the geometric means (percentage of the coefficient of variation. N/A: not applicable. ^a^Subjects in Group A did not receive abatacept. ^b^Subjects in Group B received abatacept at 14 days (serum concentration taken pre-abatacept dosing).

Subjects randomized to Group C received vaccines 2 weeks after treatment with abatacept. The mean serum concentrations observed for subjects in this group – taken 14 and 28 days after vaccinations of 12.5 μg/ml and 6.1 μg/ml, respectively – are again consistent with values at the corresponding time points in previous studies in healthy subjects.

Finally, subjects randomized to Group D received vaccines 8 weeks after treatment with abatacept. The observed mean serum concentrations of 1.3 μg/ml on study day 70 is consistent with concentration levels obtained in previous studies. Furthermore, the mean serum concentration of 0.4 μg/ml on study day 84 is consistent with concentrations that would be expected based on a half-life of approximately 14 days for abatacept.

### Antibody responses in the control group

In the control group (Group A), not all normal, healthy subjects responded fully to the two vaccines at day 14 and 28. For the tetanus toxoid, approximately 95% and 75% of subjects at days 14 and 28, respectively, achieved at least a twofold increase in antibody titers. For the pneumococcal vaccine, approximately 45–95% and 50–95% of subjects at days 14 and 28, respectively, achieved at least a twofold increase in antibody titer across all seven serotypes.

### Antibody response to tetanus toxoid

The antibody responses against tetanus toxoid vaccine, expressed as absolute titers of antibodies, are summarized in Table 3. The corresponding abatacept serum concentrations are presented in Table 2.

**Table 3:** Geometric means (percentage of the coefficient of variation) of anti-tetanus toxoid antibody titers taken 14 and 28 days after tetanus toxoid vaccination

Group	*n*	Baseline titers (U/ml)	Anti-tetanus antibody titers at 14 days post-vaccination (U/ml)	Anti-tetanus antibody titers at 28 days post-vaccination (U/ml)
Group A (vaccines alone on day 1)	20	1.6 (106)	11.4 (88)	9.3 (104)
Group B (vaccines 2 weeks before abatacept)	20	1.9 (76)	10.2 (71)	8.7 (68)^a^
Group C (vaccines 2 weeks after abatacept)	19^b^	2.3 (76)	5.9 (112)	5.6 (98)
Group D (vaccines 8 weeks after abatacept)	19^c^	2.3 (54)	9.0 (79)	7.8 (85)

^a^*n *= 19 as one subject discontinued due to an adverse event (this discontinued patient only had samples collected at baseline and day 14). ^b^Subject discontinued prior to vaccine administration on day 14 due to toxicology. ^c^Subject discontinued prior to vaccine administration on day 56 due to toxicology.

The intersubject variability in response to tetanus toxoid was large, with the percentage of the coefficient of variation ranging between 54% and 112% (Table 3). Based on the geometric mean of the antibody titers, subjects in Group B (received vaccines 2 weeks before abatacept) appeared little affected to not affected, with a lowered response of approximately 6% when compared with the control group (Group A) at 28 days after vaccination, a reduction within the variability of the assay (Table 3). For Group C subjects (received vaccines 2 weeks after abatacept), there appeared to be a lowered response of approximately 48% and 39% at 14 and 28 days, respectively, compared with Group A. Subjects in Group D (received vaccines 8 weeks after abatacept) were affected to a lesser extent, with an observed lowered response of approximately 21% and 16% at 14 and 28 days, respectively, compared with Group A.

**Figure 2:**
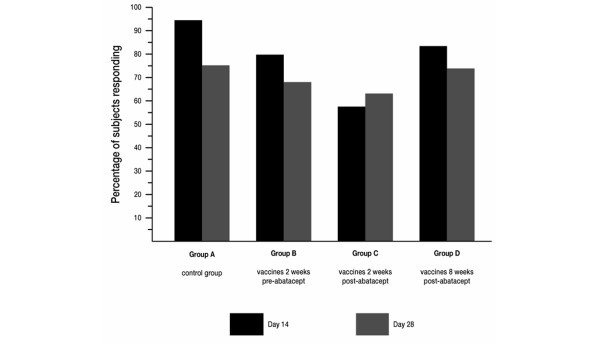
Percentage of subjects achieving at least a twofold increase in tetanus toxoid antibodies from baseline.

The percentage of subjects who mounted a response that was at least twofold from baseline is shown in Figure 2 for tetanus toxoid. Across all treatment groups, at least 60% of subjects were able to generate at least a twofold increase in antibody response after 28 days. In the control group (Group A), 75% of subjects reached this level. The responses observed at 14 and 28 days after vaccination were similar.

### Antibody responses to 23-valent pneumococcal vaccine

**Figure 3:**
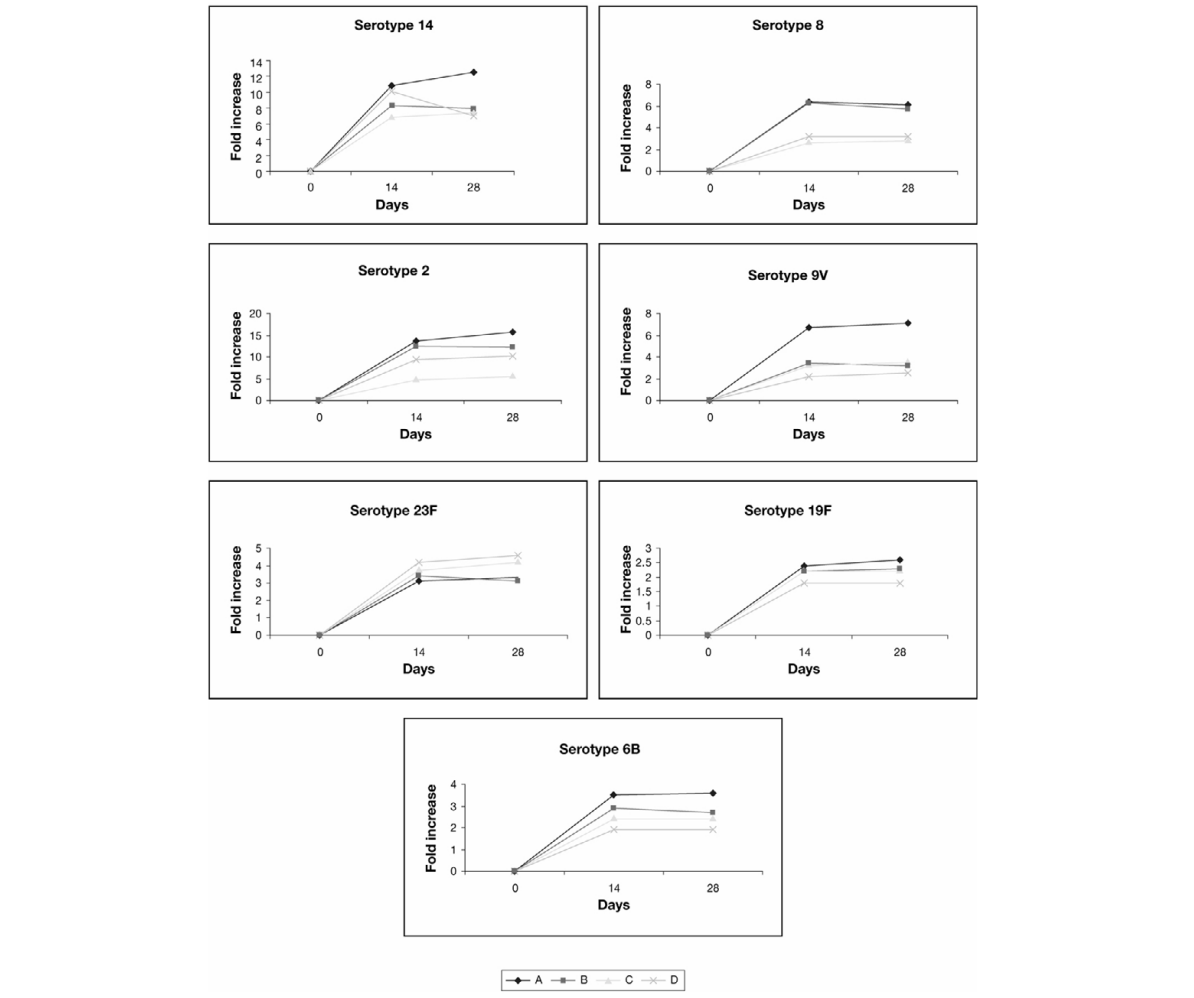
Impact of abatacept on antibody titers at 14 and 28 days after vaccination in individual pneumococcal serotypes.

Seven serotypes of 23-valent pneumococcal vaccine were chosen as a representative sample of differing immunogenic strengths of pneumococcal vaccine. Serotype 14 (the most common), serotype 8, serotype 9V and serotype 2 are the most immunogenic. Figure 3 illustrates the fold increases for the seven serotypes at days 14 and 28, respectively, and Table 4 presents the corresponding geometric mean values of antibody titers.

**Table 4:** Geometric means (percentage of the coefficient of variation) of antibody titers taken 14 and 28 days after pneumococcal vaccination

Group	*n*	Baseline (μg/ml)	14 days post-vaccination (μg/ml)	28 days post-vaccination (μg/ml)
Serotype 14				
Group A	20	1.9 (112)	20.5 (236)	23.5 (199)
Group B	20	1.9 (132)	15.5 (105)	15.4 (111)^a^
Group C	19	1.8 (201)	12.0 (195)	12.8 (250)
Group D	19	1.8 (125)	18.0 (155)	12.6 (141)
Serotype 2				
Group A	20	1.0 (163)	13.4 (113)	15.4 (110)
Group B	20	1.1 (84)	12.3 (109)	13.2 (107)^a^
Group C	19	0.8 (90)	4.1 (118)	4.8 (115)
Group D	19	0.7 (122)	7.0 (132)	7.7 (136)
Serotype 23F				
Group A	20	0.9 (101)	3.0 (112)	3.3 (113)
Group B	20	1.8 (124)	5.6 (87)	4.9 (89)^a^
Group C	19	1.2 (158)	4.5 (112)	5.1 (106)
Group D	19	1.5 (125)	6.2 (92)	6.7 (90)
Serotype 8				
Group A	20	1.5 (120)	10.1 (138)	9.6 (114)
Group B	20	2.3 (104)	12.0 (61)	10.5 (81)^a^
Group C	19	1.4 (104)	4.0 (68)	4.4 (64)
Group D	19	1.6 (74)	5.1 (144)	5.2 (106)
Serotype 9V				
Group A	20	0.9 (148)	6.1 (102)	6.4 (99)
Group B	20	1.3 (158)	4.0 (97)	3.9 (101)^a^
Group C	19	0.9 (190)	3.0 (147)	3.2 (108)
Group D	19	0.9 (119)	2.0 (107)	2.3 (106)
Serotype 19F				
Group A	20	5.3 (95)	13.0 (125)	13.9 (150)
Group B	20	10.3 (127)	19.9 (105)	19.6 (97)^a^
Group C	19	4.3 (117)	10.0 (178)	10.3 (180)
Group D	19	5.6 (75)	9.9 (89)	10.3 (88)
Serotype 6B				
Group A	20	1.6 (103)	5.9 (197)	6.1 (204)
Group B	20	3.1 (126)	7.8 (87)	7.1 (95)^a^
Group C	19	1.9 (140)	4.6 (147)	4.6 (141)
Group D	19	1.8 (114)	3.5 (159)	3.5 (151)

^a^*n *= 19.

**Figure 4:**
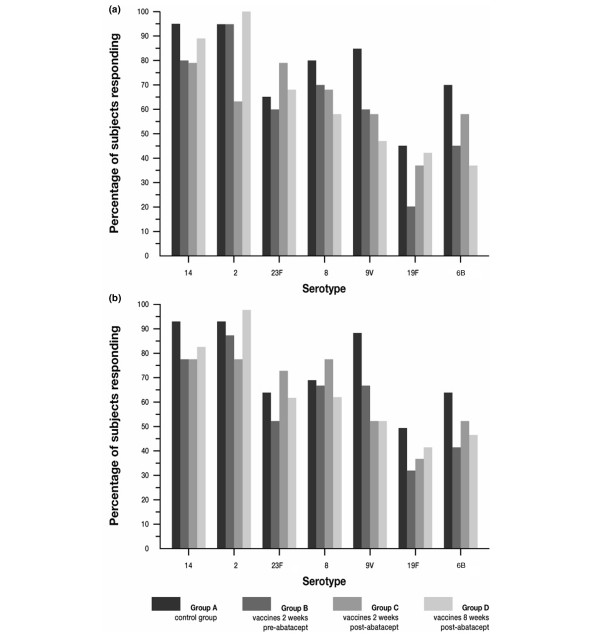
Percentage of subjects achieving at least a twofold increase in antibody titers for individual pneumococcal serotypes. **(a) **14 days after vaccination and **(b) **28 days after vaccination.

As with the response to tetanus toxoid, variable response rates were obtained in the study subjects across individual serotypes. The percentages of subjects in all treatment groups achieving a positive response to the different serotypes at 14 and 28 days after vaccination are illustrated in Figure 4a and 4b, respectively. In general, and as expected, the highest responses were observed for serotypes 14 and 2. The apparent decrease in vaccination response in subjects who were in Group B cannot be accurately evaluated because of the higher baseline values obtained in these subjects, a known cause of reduced relative responses. This randomization variability is further illustrated by the fact that responses in Group B subjects appeared decreased even at day 14, prior to the administration of abatacept. In subjects of Groups C and D, however – those who were vaccinated after abatacept – lower average titers on days 14 and 28 were recorded for all serotypes, except serotype 23F (Table 4). The decrease in antibody response in Group C subjects at 14 and 28 days after vaccination ranged from 22% to 69% and from 24% to 68%, respectively. Similarly, the decrease in antibody response for subjects in Group D determined at 14 and 28 days after vaccination ranged between 12% and 67% and between 25% and 64%, respectively. No correlation between the immunogenicity of the serotype of the pneumococcal vaccine and the reduction in response was observed.

**Figure 5:**
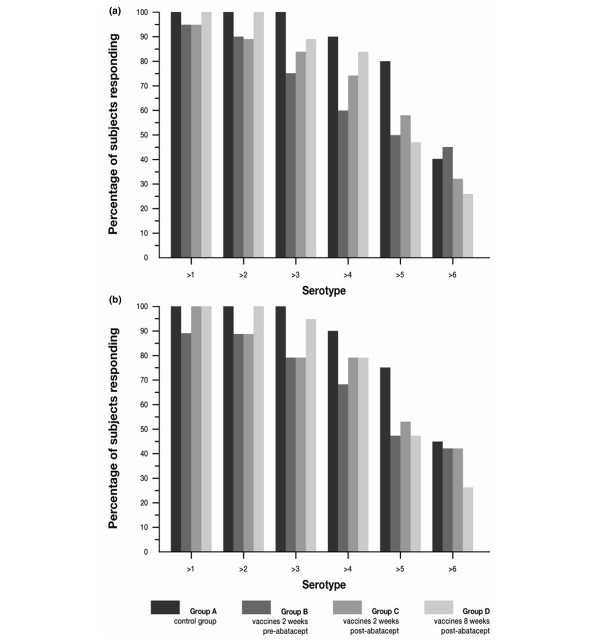
Number of pneumococcal serotypes to which subjects responded. **(a) **14 days after vaccination and **(b) **28 days after vaccination.

Figure 5a,b summarizes the number of serotypes to which subjects responded with at least a twofold increase over baseline at 14 and 28 days after vaccination, respectively. More than 90% of subjects in all treatment groups responded to at least one serotype, over 70% of subjects responded to at least three different serotypes, and approximately 25% of subjects responded to at least six different serotypes by day 14 (Figure 5a) and by day 28 (Figure 5b).

## Discussion

The purpose of this study was to investigate the effect of abatacept on the antibody response in healthy subjects prior to initiating studies in RA patients. Tetanus toxoid vaccine and the 23-valent pneumococcal vaccine were used to assess the impact of abatacept on a memory response to a T-cell-dependent protein antigen and to a less T-cell-dependent polysaccharide antigen, respectively. Finally, the correlation of any effect regarding the timing of abatacept administration relative to the administration of each vaccine was evaluated.

The geometric mean titers were reduced for both vaccines, suggesting that abatacept does blunt the immune response to these vaccines. This effect on the response occurred to differing extents among the groups. For the tetanus toxoid vaccine, Group C subjects (vaccines 2 weeks after abatacept) appeared to be the more affected of the three treatment groups, compared with Group A subjects (control group). Subjects in Group D (vaccines 8 weeks after abatacept) were affected to a lesser extent than those in Group C, and Group B subjects were the least affected.

For the 23-valent pneumococcal vaccine, there appeared to be lower titers for all serotypes, except serotype 23F, in subjects of Group C (vaccines 2 weeks after abatacept) and of Group D (vaccines 8 weeks after abatacept). The apparent decrease in vaccination response in subjects in Group B cannot be accurately evaluated because of the higher baseline values obtained in these subjects.

While abatacept reduced the response (geometric mean titers) of the two vaccines, it did not significantly inhibit the ability of healthy subjects to develop at least a twofold response to either the tetanus toxoid or 23-valent pneumococcal vaccine. Overall, across all treatment groups, >60% subjects were able to generate at least a twofold increase in antibody response to tetanus toxoid after day 28, and over 70% of subjects in all treatment groups responded to at least three serotypes of the pneumococcal vaccine; in addition, approximately 25% of all treated subjects responded to at least six serotypes – an expected and normal response in healthy subjects [18,19].

The role of abatacept in the reduction of the geometric mean titers is supported by the relationship between serum levels of abatacept present at the time of vaccination and the degree of inhibition of the humoral response. The most affected group in this study was Group C (vaccine 2 weeks after abatacept). In this group, the highest abatacept levels (and presumably a higher degree of co-stimulation blockade) were observed at the time of vaccination. By contrast, the observed mean serum concentration for Group D subjects (vaccine 8 weeks after abatacept) was very low at the time of vaccination and was less affected. Group B subjects (vaccine 2 weeks before abatacept) appeared to be least affected, at least for the tetanus toxoid. This may be due to the fact that a peak antibody concentration in a normal primary immune response is achieved at around 2 weeks [12], and in Group B subjects there was presumably a pool of B cells that had completed their differentiation into antibody-secreting plasma cells before abatacept was administered.

Abatacept prevents the activation of naive T cells by inhibiting the second signal required for their co-stimulation. This signal is mediated by CD80 and CD86, which is expressed on antigen-presenting cells, and by CD28, which is expressed on T cells. Abatacept may also reduce the activation of memory T cells (although to a lesser extent than for naïve T cells) [20]. This is consistent with a reduced response against tetanus toxoid. The inhibition of the CD80/CD86:CD28 co-stimulatory signal may also potentially prevent the T-cell 'help' needed for optimal differentiation of CD80/CD86-expressing B cells into plasma cells, which ultimately secrete antibodies. This inhibition of B cell–T cell help may be a reason for the reduced antibody response to thymus-independent polysaccharide antigens such as those contained in the pneumococcal vaccine – responses that cannot be considered completely T-cell independent since they are enhanced by T-cell help [9,11]. Finally, since abatacept inhibits one of several mediators of co-stimulation, the partial inhibition observed here is likely to reflect the redundancy of the co-stimulation mechanism.

This study analyzed the response in healthy volunteers with a normal immune system to a single dose of abatacept. Future studies are needed to determine the optimal timing of vaccination in RA patients receiving abatacept continuously, possibly with other concomitant disease-modifying antirheumatic drugs such as methotrexate.

## Conclusion

This study suggests that abatacept blunts the effectiveness of the immune response, but does not significantly inhibit the ability of healthy subjects to develop a clinically significant or positive immune response (at least a twofold increase above baseline) to both tetanus toxoid and 23-valent pneumococcal vaccines.

## Abbreviations

AE = adverse events; ELISA = enzyme-linked immunosorbent assay; i.m. = intramuscular; i.v. = intravenous; RA = rheumatoid arthritis; TNF = tumor necrosis factor.

## Competing interests

LT, GV, RR and MC are employees of Bristol-Myers Squibb (Princeton, NJ, USA) and own stock options. FL is an ex-employee of Bristol-Myers Squibb (Princeton, NJ, USA) but no longer possesses stock in Bristol-Myers Squibb or has any other financial disclosure.

## Authors' contributions

LT, FL, GV and MC were involved in the development of the study design. RR developed the statistical plan and performed the analyses. LT also participated in the pharmacokinetic analysis and design. All authors were involved in the drafting and revision of the manuscript. All authors read and approved the final manuscript.
